# Update on Fc-Mediated Antibody Functions Against HIV-1 Beyond Neutralization

**DOI:** 10.3389/fimmu.2019.02968

**Published:** 2019-12-18

**Authors:** Bin Su, Stefania Dispinseri, Valeria Iannone, Tong Zhang, Hao Wu, Raphael Carapito, Seiamak Bahram, Gabriella Scarlatti, Christiane Moog

**Affiliations:** ^1^Center for Infectious Diseases, Beijing Youan Hospital, Capital Medical University, Beijing, China; ^2^Beijing Key Laboratory for HIV/AIDS Research, Beijing, China; ^3^Viral Evolution and Transmission Unit, Division of Immunology, Transplantation, and Infectious Diseases, San Raffaele Scientific Institute, Milan, Italy; ^4^INSERM U1109, LabEx TRANSPLANTEX, Fédération Hospitalo-Universitaire (FHU) OMICARE, Fédération de Médecine Translationnelle de Strasbourg (FMTS), Université de Strasbourg, Strasbourg, France; ^5^Vaccine Research Institute (VRI), Créteil, France

**Keywords:** HIV-1, antibody functions, non-neutralizing antibodies, FcR-mediated inhibition, ADCC

## Abstract

Antibodies (Abs) are the major component of the humoral immune response and a key player in vaccination. The precise Ab-mediated inhibitory mechanisms leading to *in vivo* protection against HIV have not been elucidated. In addition to the desired viral capture and neutralizing Ab functions, complex Ab-dependent mechanisms that involve engaging immune effector cells to clear infected host cells, immune complexes, and opsonized virus have been proposed as being relevant. These inhibitory mechanisms involve Fc-mediated effector functions leading to Ab-dependent cellular cytotoxicity, phagocytosis, cell-mediated virus inhibition, aggregation, and complement inhibition. Indeed, the decreased risk of infection observed in the RV144 HIV-1 vaccine trial was correlated with the production of non-neutralizing inhibitory Abs, highlighting the role of Ab inhibitory functions besides neutralization. Moreover, Ab isotypes and subclasses recognizing specific HIV envelope epitopes as well as pecular Fc-receptor polymorphisms have been associated with disease progression. These findings further support the need to define which Fc-mediated Ab inhibitory functions leading to protection are critical for HIV vaccine design. Herein, based on our previous review Su & Moog Front Immunol 2014, we update the different inhibitory properties of HIV-specific Abs that may potentially contribute to HIV protection.

## Introduction

**Figure 1:**
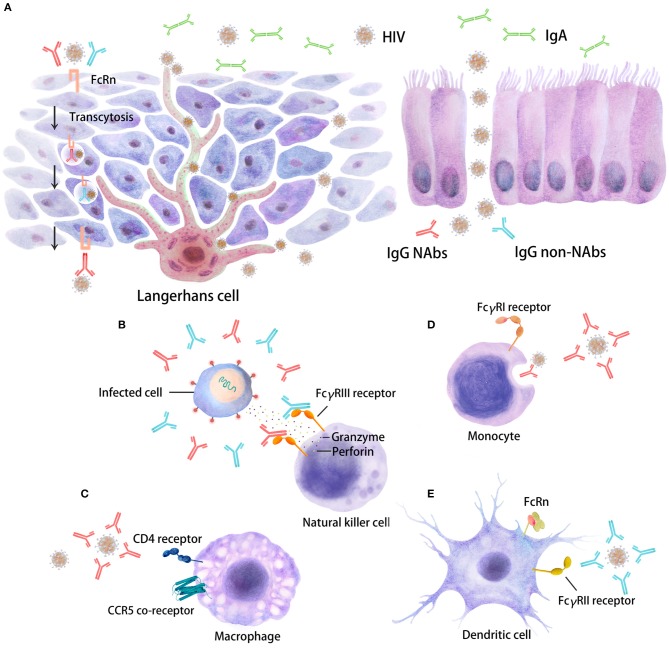
Distinct inhibitory activities of Abs in HIV target cells. The interaction of Fc regions with Fc gamma receptors (FcγRs) on immune cells, such as dendritic cells (DCs), macrophages, and natural killer (NK) cells, will trigger several antiviral immune responses, including immune activation, which are able to restrain HIV replication. Five major Ab responses against HIV infection that can occur as a result of IgG neutralizing Abs (depicted in red), IgG non-neutralizing inhibitory Abs (depicted in blue) or IgA (in green) are shown. **(A)** There are two types of stratified epithelium and columnar epithelium. The binding of virus/Ab immune complexes to FcRn at the surfaces of Langerhans cells may lead to transcytosis through genitorectal mucosal surfaces; **(B)** FcγRIII-mediated antibody-dependent cellular cytotoxicity (ADCC); **(C)** neutralizing antibody (NAb)-mediated activity; **(D)** phagocytosis mediated by NAbs/non-NAbs through FcγRI binding; **(E)** FcγRII-mediated non-neutralizing inhibition; some FcγR/Fc interactions may also enhance HIV entry and infection. These different inhibitory activities can occur at mucosal sites beneath the mono- or pluri-stratified epithelial layers where HIV target cells reside.

Currently, sexual transmission is the major route for human immunodeficiency virus (HIV) infection and contributes to 80% of newly diagnosed cases worldwide. This statistic implies that the virus crosses the mucosal barrier to reach and infect HIV target cells (1) and that an effective vaccine needs to induce an immune response that acts rapidly at mucosal sites. Neutralizing antibodies (NAbs), which can be IgG or secretory IgA, are certainly desired for blocking HIV transmission and have been shown to be highly effective at preventing infection through this route (Figure 1) (2, 3). During the last decade, a whole new series of broadly neutralizing Abs (bNAbs), which are NAbs with exceptional potency and breadth, have been isolated (4–6) and have efficiently protected humanized mice and non-human primates (NHPs) from experimental challenge. Some of these bNAbs are undergoing testing in human clinical prevention and therapeutic trials (6–14). However, bNAbs that display these features have very specific characteristics. Indeed, bNAbs exhibit uncommonly long complementarity-determining loops and extensive somatic hypermutation, which requires a long maturation process (6, 15–17). In turn, bNAbs are developed by only 10–30% of HIV-infected individuals (6, 17–20), and attempts to induce them by vaccination have encountered extreme difficulties (17, 21).

An increasing body of evidence suggests that Ab functions mediated by the Fc domain may play a role in protection against infections (22–27). Interestingly, the moderately protective effects observed in the RV144 HIV-1 vaccine trial were achieved in the absence of detectable NAbs, suggesting an important role for Fc-mediated functions in protection (28, 29). Fcγ receptor (FcγR)-mediated Ab responses, which lead to phagocytosis, aggregation, complement inhibition, Ab-dependent cellular cytotoxicity (ADCC), Ab-dependent cellular phagocytosis (ADCP), and Ab-dependent cell-mediated virus inhibition (ADCVI), have been shown to decrease HIV replication and may therefore substantially contribute to HIV protection (22–25, 27). This review focuses on the importance of Ab Fc-mediated functions in preventing HIV-1 infection and highlights their possible relevance for the development of new vaccine strategies.

## Antibody Responses During HIV Infection

Significant efforts have been made over the past two decades to deepen the understanding of the role of the humoral immune response in HIV infection and to foster the development of vaccine strategies to control viral replication (30). The acquisition of HIV-specific IgG Abs appears within the first 2 months (31, 32) and evolves during the course of infection. In the neonatal period, the early serological response to infection in infants is obscured by the presence of transplacentally acquired maternal HIV Ab. The amount of immunoglobulin to HIV-1 and the number of HIV-1 antigens recognized increases with age (31, 33). The emergence of Ab responses to the viral envelope during HIV infection can generally be divided based on the timing of their appearance and their functions (34, 35). These responses include the following:

1. A non-neutralizing inhibitory Ab (non-NAb) response directed at immunogenic epitopes that develops in all individuals soon after infection. Typically, this Ab response is directed first against viral gp41 following gp120/CD4 binding (34, 36) and soon thereafter against the V3 loop of gp120 (37). These Abs have a low impact on the virus and plasma viral load because they may have limited interactions with functional Env trimers (38). However, they may play an important role in protection via Fc-mediated effector functions, although the exact mechanism remains to be elucidated (24). Holl et al. reported that polyclonal sera with non-neutralizing activity inhibit HIV replication in macrophages by the phagocytosis of immune complexes bound to FcR expressed on cells (27). In a clinical trial called RV144, non-NAbs exhibiting *in vitro* inhibitory functions were described and associated with decreased HIV acquisition (39, 40).
2. An HIV strain-specific NAb response targeting Env epitopes that are expressed on the native trimer, which is directed toward viruses present earlier in infection and is detected within the first year after seroconversion (20, 30). The NAbs recognizing Env epitopes via their Fab fragments may block HIV entry. An autologous NAb response is observed *in vitro* in the absence of additional factors, such as FcRs or complement, and is mainly a result of the blocking of virus-cell interactions (41). Such NAbs will encounter challenges, as they have to cope with a staggering level of viral diversity. Continuous viral escape from NAbs will occur as a consequence of single amino acid substitutions, insertions, and deletions, and through an “evolving glycan shield,” in which shifting glycans prevent access by Abs to their cognate epitopes (38). Nonetheless, these autologous neutralizing responses may display additional effector functions involving Fc-mediated contributions to the decreases in the viral load detected within the first months after HIV acquisition (42).
3. An Ab response capable of neutralizing a wide range of viral isolates that develops 2–5 years after seroconversion (43–46). However, this bNAb response occurs in only a minority of patients and is associated with increased HIV replication and diversity, although bNAbs can sometimes be detected in subjects that control HIV (16, 47, 48) or in chronically HIV-infected individuals (19). Ultimately, the virus will escape from bNAbs. Notably, bNAbs have also demonstrated efficient Fc-mediated inhibitory function in addition to neutralization.

Therefore, during the course of HIV infection, the Ab response evolves, leading to complex polyfunctional activities that may certainly impact the course of HIV disease. The specific role of bNAbs in disease evolution and the potential contributions of other inhibitory functions have not been firmly demonstrated.

## New Generation of bNAbs

Thanks to major improvements in Ab isolation technologies, more than 100 HIV-1-specific bNAbs with remarkable potency against a wide variety of HIV subtypes have been developed (4, 6, 8, 9, 17, 43, 44, 49–53). The breadth of viral recognition and the antiviral potency of bNAbs can be classified according to their preferential target on the Env spike (4, 26, 49, 54, 55). Passive transfer of bNAbs performed in macaques has shown their remarkable capacity to protect non-human primates (NHPs) from experimental simian-HIV (SHIV) challenge when administered via different routes and modes (a single high dose or repeated low doses administered by the intravenous, rectal or vaginal route) (56–59). Interestingly, recent studies have demonstrated that such protection was not necessarily sterilizing, as was previously thought. Indeed, a few infected cell foci were detected 1–3 days after experimental challenge (60, 61), which intriguingly disappeared leading to complete protection. These results strongly suggest that protection is not solely due to neutralization of the virus particles and that Fc-mediated inhibitory function leading to the lysis of HIV-infected cells by bNAbs participate in this protection (62–64).

Although the newer bNAbs react with more than 90% of circulating HIV-1 strains when tested *in vitro*, at present, no single bNAb potently neutralizes all HIV strains. Therefore, a combination of two or more bNAbs would be desirable to cover the entire range of viral strains encountered *in vivo* (6, 7, 65, 66). In this regard, bi-specific and even tri-specific Abs targeting multiple HIV-1 Env epitopes were developed recently to increase Ab breadth and potency (67–70). These new bNAbs were characterized following the sequencing of their Fab heavy and light chains and were further reconstituted with an IgG1 heavy chain to form the Ab Fc domain. Most bNAbs have high levels of somatic hypermutation, including amino acid in-frame substitutions (71), frequent in-frame insertions and deletions (72), and genetic bias in the Ig heavy chain variable region (IGHV) (73). Modifications of the FcR domain were introduced in some of these bNAbs to increase their stability and persistence *in vivo* and to potentially allow long-lasting activity and less frequent administration (23, 74–76).

## Antibody Isotypes and Their Subclasses

**Figure 2:**
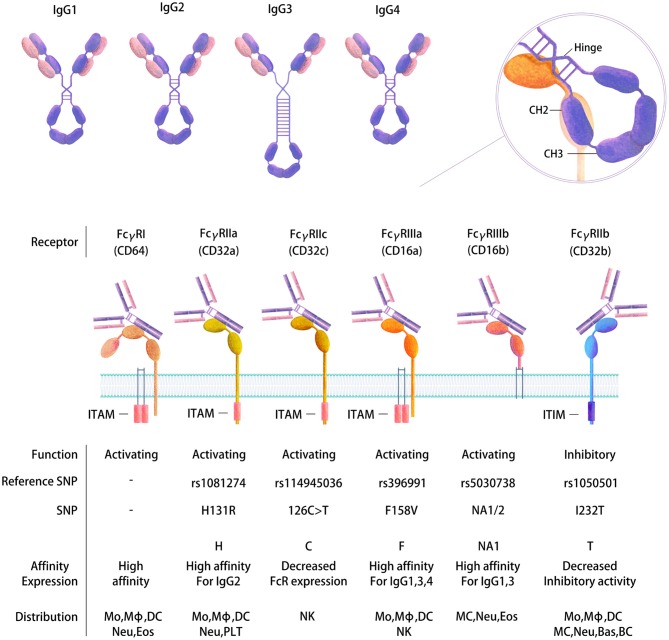
Human FcγR gene polymorphisms and the expression and affinity of IgG subtypes. Four IgG subtypes are present in human serum that have distinct structures and functions (top). FcγRs belong to the Ig receptor superfamily and comprise two or three extracellular Ig domains that mediate IgG binding. Differential immune regulatory effects are produced depending on binding to FcRs. Activating or inhibitory functions occur based on the presence of an intracellular cytoplasmic domain ITAM or ITIM motif that transduces an immunostimulatory or inhibitory signal, respectively, following receptor cross-linking. Binding of the Fc to the receptors is mediated at the CH2-CH3 interface following a conformational change (right). The diversity of FcγRs is further increased by SNPs in their extracellular domains, which in turn affect the expression of FcRs and their binding affinity and function (bottom). Mo, Monocyte; Mϕ, Macrophage; DC, Dendritic cell; MC, Mast cell; Neu, Neutrophil; Bas, Basophil; Eos, Eosinophil; NK, Natural killer cell; BC, B cell; PLT, Platelet.

Upon B-cell activation by immune complexes, an HIV-specific Ab response will be generated that induces the production of immunoglobulins (Ig) of different types and isotypes (77, 78). These types and isotypes differ in their related Ig heavy chains, which therefore impacts the Fc-mediated function of the Abs. Four IgG subtypes are present in healthy adult serum: IgG1 (60–72%), IgG2 (20–31%), IgG3 (5–10%), and IgG4 (<4% of the total main IgG subclasses) (79) (Figure 2), whereas the gp120-specific IgG subclass distribution and IgA/IgM distribution are generally as follows: IgG1>IgG2 = IgG4>IgG3 and IgA>IgM (80). Indeed, diverse types (IgM, IgG, and IgA) and IgG subtypes will be induced by HIV in plasma and other body fluids, such as cerebrospinal fluid, saliva and genital secretions (80). The Ig subclasses will vary according to the type of infection, the related inflammatory stage and pathogen localization (81). Therefore, the isotype distribution following infection remains to be established. Some studies have proposed that Abs against gp41 are mainly IgG1 rather than other IgG subtypes or IgA or IgM classes (80, 82), whereas others reported gp41-specific Abs of other subclasses (83, 84). The functional role of these different isotypes also needs to be better characterized. It has been proposed that Env-specific gp41 IgM and IgG Ab responses have little effect on the control of the acute phase of viral replication (36). IgG1 and IgG3 Abs are highly active against viral infection, and IgG3 Abs appear first during the course of infection (85), whereas the role of the IgG2 subclass is not known. This isotype is mainly induced by bacterial capsular polysaccharide antigens (79, 86). Moreover, the IgG subclass prevalence has been reported to change over time. For example, gp120-specific IgG1 levels remain constant during the first 6 months after infection, whereas the level of gp120-specific IgG3 peaks after 1 month and then declines (84). Another example is gp140-specific IgG2 and IgG3 responses, which do not occur simultaneously in HIV-1-infected individuals (87). Isotype switching may largely impact Ab functionalities linked to Fc affinity toward Fc receptors (22, 78).

The Ab isotype/subclass recognition of Env epitopes is associated with certain disease courses, symptoms and responses to medication. Assessment of the IgG subclass distribution in the plasma of HIV-1-infected patients enrolled in a French prospective asymptomatic long-term (ALT) cohort showed that in contrast to the IgG1 titers, the IgG2 titers directed against HIV-1 Env gp41 and anti-p24 Abs were correlated with and were highly predictive of decreased viral loads and slower disease progression, especially long-term non-progression (88). This observation was confirmed recently (89, 90). Moreover, Martinez et al. found that in addition to HIV-1-specific CD4^+^ Th1 cell responses, anti-gp41 IgG2 was the best predictor of long-term non-progression (91). In the RV144 vaccine trial (29, 92), the serum Env-specific IgA level was correlated with an increased risk of HIV-1 infection, which was potentially due to monomeric circulating IgA competing with IgG and interfering with the ability of IgG to mediate ADCC and ADCP (28). The presence of IgG3 against the Env V1V2 region was correlated with a lower risk of HIV-1 infection (29, 93). Interestingly, IgG3 appeared to neutralize HIV more efficiently *in vitro* than IgG1 (94). Moreover, the plasma IgG1 and IgG2 anti-HIV-1 p24 levels were inversely correlated with the plasma HIV RNA levels in viremic HIV patients (95).

In addition to isotype switching, the glycosylation profile of Ig changes during infection. Agalactosylation and afucosylation were more common in HIV-specific Abs among patient with spontaneous control of HIV and were linked to enhanced NK cell activity (96). The modifications of specific glycan groups determine the functional properties of Abs.

Taken together, these different results suggest that dynamic Ab subclasses/isotypes, posttranslational modifications, and glycosylation will impact disease progression. A comprehensive interrogation of the extensive biological diversity in naturally or experimentally protected subjects may provide insights critical for guiding the development of effective vaccines and Ab-based therapies.

## Fc-Receptor: The Cellular Counterpart for Antibody-Mediated Responses

Each Ig isotype binds to specific Fc receptors, which in humans are the high-affinity receptors Fcα/μR for IgA and IgM, FcμR for IgM, FcαRI for IgA, FcεRI for IgE and FcγRI and neonatal Fc receptor (FcRn) for IgG and the low-affinity receptors FcεRII for IgE and FcγRII and III for IgG (97) (Table 1). Special attention has been paid to genes encoding the Fcγ receptors, since they bind the constant domain of IgG, which is the major Ab type induced by the host response following viral or bacterial infection (98). Human cells express three FcγRIIs (A–C) and two FcγRIIIs (A and B). All human FcγRs except FcγRIIB signal through an immunoreceptor tyrosine-based activating motif (ITAM), whereas FcγRIIB delivers inhibitory signals through an immunoreceptor tyrosine-based inhibitory motif (ITIM) (99) (Figure 2). The diversity of human FcγRII and III is further increased by the presence of single nucleotide polymorphisms (SNPs) in their extracellular domains, the most studied of which are H131R in the FcγR gene *FCGR2A* (100), 126C>T in *FCGR2C* (99), F158V in *FCGR3A* (101), and NA1/2 in *FCGR3B* (102) (Figure 2). FcγRIIC has an unusual structure and is generated by the unequal crossover of FcγRIIA and FcγRIIB (99, 103). *FCGR2C* in FcγRIIC (126C>T) shares the extracellular sequence of *FCGR2B* but signals through the ITAM, similar to *FCGR2A*.

**Table 1:** Fc receptors and their corresponding Ig binding in humans.

**Receptor**	**Fcα/μR**	**FcμR**	**FcαRI**	**FcεRI**	**FcεRII**	**FcγRI**	**FcγRIIa**	**FcγRIIb**	**FcγRIIc**	**FcγRIIIa**	**FcγRIIIb**
Human Ig	IgA/IgM	IgM	IgA	IgE	IgE	IgG	IgG	IgG	IgG	IgG	IgG
Affinity for monomer Ig	^*^N.D.	^*^N.D.	Low	High	Low	High	Low	Low	Low	Medium	Low

* *N.D., not determined*.

Importantly, the different FcR polymorphisms in the host need to be taken into consideration when analyzing the FcR-mediated functions of Abs. FcγR SNPs will impact both binding to the complementary Fc portion of the Abs and on the other side the expression and activation state in cells (Figure 2). Indeed, increasing evidence suggests that FcγR SNPs impair receptor expression on DCs, which in turn influences the risk of HIV infection and vaccine efficacy (104). Similarly, the FcγRIIIA polymorphism appears to modify NK cell activation and, as a consequence, ADCC activity (105). Specific polymorphisms in the *FCGR2A* (encoding Arg at position 131) and *FCGR3A* (encoding Phe at position 158) gene loci have been associated with decreased HIV acquisition (106). The latter SNP leads to the increased binding capacity of Abs for FcγRIIIA, which is the receptor involved in ADCC, suggesting that vaccine efficacy may be related to the increased efficacy of this function. More recently, Li et al. found that a tagged SNP (rs114945036) in *FCGR2C* (126C>T) was significantly associated with protection against infection with the HIV-1 AE subtype strain in the RV144 vaccine clinical trial. The direct effect of this SNP is not well-documented, although the authors proposed that it may lead to an FcR with an atypical FcR protein sequence, thereby modifying FcR expression or accessibility on cells (99). Interestingly, FcγRIIC has been reported to mediate ADCC and may play a role in anti-HIV-1 Ab neutralizing activity similar to that of FcγRIIB (103, 107–109).

Furthermore, although it has not yet been thoroughly investigated, there is evidence that FcγR polymorphisms are associated with mother-to-child transmission of HIV. Mothers with the FcγRIIIa-158V allele have enhanced binding affinity for IgG and ADCC capacity, which reduces the susceptibility of their fetuses to HIV infection and significantly reduces the chance of mother-to-child transmission during both the intrapartum and *in utero* periods compared with the FcγRIIIa-158F allele (110, 111).

These studies showing the significant role of FcγR polymorphisms strongly suggest that the Fc-driven function induced by vaccination may play a role in HIV protection. However, whether specific FcR polymorphisms are also involved in the control of HIV replication is not clear. One study did not detect significant differences when comparing the genotype profiles of *FCGR2A* and *FCGR3A* (polymorphisms H131R and V158F, respectively) in 73 patients in which HIV infection was controlled with those in patients who progressed to disease (112). Conversely, another study showed that the combination of these two SNPs was significantly associated with HIV progression in 53 patients who progressed compared with 43 patients in which HIV infection was controlled (113). Additional studies will be needed to define the roles of these SNPs in HIV replication and disease.

Importantly, the Fc regions of Abs contain a binding epitope for FcRn (neonatal FcR), which is responsible for the extended half-life, placental transport, and bidirectional transport of IgGs or immune complexes through the mucosal layer (Figure 1) (75, 114–116). FcRn is also expressed in myeloid cells, where it participates in both phagocytosis and antigen presentation together with the classical FcγR and complement. The relevance of this receptor in HIV infection has not been defined. However, this characteristic was largely exploited by modifying Fc regions in monoclonal Abs for use in the treatment of cancer or HIV infection.

## Fc-Mediated Ab Functions

Abs with Fc-mediated inhibitory activities, such as ADCC, ADCP, or aggregation, in addition to neutralizing activity have been detected at all stages of HIV disease. These inhibitory functions involve the Fc domains of Abs as well as the Fab domain. Therefore, both the Ab isotype and FcR expression on effector cells will be determinants of these functions (Figure 1).

### Antibody-Dependent Cellular Cytotoxicity

ADCC is a complex but potent Fc-mediated effector function that is involved in the clearance of malignant or infected cells. In the latter process, ADCC eliminates virus-infected cells through mediating cooperation between innate and acquired immunity (117–119). Specifically, Abs act as a bridge between an infected target cell and an effector cell; the Fab domain binds to a specific viral antigen expressed by the infected cell, and the Fc domain binds to FcγR expressed on the surface of the effector cell (i.e., NK cells, monocyte/macrophages, and neutrophils) (120–122). As a result of this interaction, effector cells release perforin and granzymes, leading to death of the Ab-bound infected target cells. Several studies have shown an association between ADCC and slower disease progression in NHPs and humans (29, 123–126), highlighting the importance of ADCC *in vivo* (25, 26, 126–128). In the NHP model, ADCC was associated with protection from infection by a pathogenic virus (129). Interestingly, Abs directed against the V2 epitope were found to efficiently exhibit ADCC activity *in vitro* (130, 131). Similarly, non-NAbs targeting the V2 region of Env were associated with a decreased risk of HIV acquisition in the RV144 vaccine trial in Thailand (28, 29, 92). The relevance of ADCC was also demonstrated for mother-to-child transmission (MTCT), where the presence of ADCC-mediating Abs was associated with improved clinical status, delayed disease progression in infants (132) and a reduced risk of infection through breastfeeding (133).

In-depth studies are required to determine how Abs clear HIV-1-infected cells, including the investigation of epitopes recognized by ADCC-mediating Abs, naturally occurring Fc domains on ADCC-mediating Abs and Fc receptors on physiologically relevant effector cells (134). Further studies will be required to determine how to elicit the appropriate combinations of Abs and effector cells in the desired locations by vaccination. Because ADCC is a complex, multilayered process, the detection of this process using *in vitro* assays is challenging. Numerous assays have been developed to analyze ADCC activity *in vitro*. These assays differ in their use of various effector and target cell types (cell lines or primary cells), antigens (Env or whole virus), and read-outs (binding, effector cell activation, granzyme release or infected cell lysis). As a consequence, the results obtained from each ADCC assay will reflect the different aspects and factors involved in each step (135).

First, Abs bind a specific epitope on target cells to mediate ADCC. This step can occur either during early events of virus binding to target cells or at a later step when viral epitopes are expressed on infected cells (121, 128, 136). As a consequence, different Env conformations can be involved according to the infection stage, and therefore the recognition of different specific epitopes by Abs may impact the ADCC results (137). Moreover, caution needs to be taken to ensure that the identified Abs effectively target infected cells and not uninfected cells that have captured HIV Env via their CD4 receptor (138–140). Therefore, identifying the viral epitopes on infected cells involved in ADCC is critical. The number of viral epitopes targeted by ADCC activity seems to be higher than that targeted by neutralization. Indeed, numerous non-NAbs recognizing non-functional spikes on the viral surface or specific conformations of epitopes expressed on infected cells were shown to mediate ADCC without displaying neutralizing activity. We may very well-propose that Abs mediating ADCC may be complementary to bNAbs in potentiating the inhibitory activity of an HIV vaccine.

Second, the Fc domain of an Ab binds to the FcR expressed on effector cells. This binding is dependent on one side of the Ab heavy chain sequence. Interestingly, the Fc domain sequence is influenced by B-cell activation, the recognized epitope and intrinsic donor variability and therefore varies according to the isotype and gene rearrangement. Notably, newly developed monoclonal Abs were generated by their reconstruction with a same IgG1 heavy chain therefore all expressing an identical Fc domain. These constructs provided the opportunity to analyze ADCC while maintaining a constant Ab Fc domain. However, we must remember that these bNAbs do not reflect the large spectrum of variability in the Ab Fc domain *in vivo*.

Third, the function of Abs is dependent on the polymorphisms and expression of the FcRs on effector cells. FcR expression is regulated in different cell populations according to their localization, maturation stage, and genotype. For example, NK cells from healthy donors are usually used as effector cells to test ADCC *in vitro*. However, little is known about how the distribution of FcR expression on NK cells varies in different tissues and among individuals. This phenomenon may impact vaccine responses, HIV transmission and disease progression. Moreover, the combination of specific HLA and killer immunoglobulin-like receptor (KIR) expression on NK cells was shown to play a role in protection against infection and elite control (141–145). Taken together, the results suggest that all primary NK cell variables may influence ADCC outcomes *in vitro* if not adequately standardized. This may be overcome by using an NK cell line, albeit at the expense of the physiological variability of FcRs *in vivo*.

Finally, the assays currently being developed in the field use distinct read-outs (24, 134, 146). The detection of the lysis of HIV-infected cells is the ultimate physiologically relevant read-out, but this outcome is technically highly challenging to measure. More straightforward read-outs, such as detection of FcR triggering, are being proposed, but whether these indirect detections methods effectively reflect ADCC function is not clear. Further investigations are urgently required to precisely define the important epitopes and to determine how to efficiently trigger effector cells to achieve the *in vivo* destruction of infected cells.

### Antibody-Dependent Cellular Phagocytosis

Abs can also eliminate opsonized pathogens through phagocytosis via the engagement of FcRs expressed by cells of the innate immune system, including monocytes, macrophages, neutrophils, dendritic cells (DCs), and mast cells (27, 147–149). Phagocytosis is important for pathogen clearance by direct lysis or antigen presentation and innate immune cell activation with consequent pathogen elimination. The detection of ADCP *in vitro* leads to efficient inhibition of HIV replication in infected cells (27, 148, 149) and is associated with protection from repeated intrarectal challenge with SHIV-SF162P3 or SIVmac251 in immunized rhesus macaques (150–152). ADCP mediated by IgG3 Abs was elicited in recipients of the RV144 vaccine (153). However, the epitope specificity of Abs mediating ADCP still needs to be investigated. Musich et al. demonstrated that anti-V2 monoclonal Abs mediated ADCP activity in a dose-dependent manner similar to anti-V3 and CD4bs monoclonal Abs against clade B gp120 (154) but displayed increased activity against clade C gp120 compared to anti-V3 and anti-CD4bs monoclonal Abs, suggesting the broader recognition of exposed epitopes (154); this may also have been due to V2 epitopes being more conserved between clade B and C than V3 epitopes. Moreover, the role played by the cell type that mediates ADCP should also be defined. Current *in vitro* ADCP assays mainly use cell lines that may largely reduce the physiological relevance of these *in vitro* assays. However, recently, phagocytosis mediated by macrophages or activated neutrophils in human mucosal and lymphoid tissues was proposed to play a significant role in protection from infection (155). Thus, this type of assay requires further development to better define the *in vivo* role of ADCP function.

### Antibody-Dependent Cell-Mediated Virus Inhibition

ADCVI involves a combination of different FcγR-mediated antiviral activities that occur when an Ab bound to a virus-infected target cell engages FcγR-bearing effector cells, such as NK cells, monocytes or macrophages (156). *In vitro* virus inhibition assays partly measure target cell death mediated by ADCC and partly measure non-cytolytic mechanisms of HIV inhibition due to β-chemokine release from effector cells or the phagocytosis of immune complexes (27, 42, 157).

Overall, Fc-mediated function was found to actively contribute to Ab inhibitory activities. The combination of these activities was found to be associated with protection (158).

The involvement of Fc-mediated activity in HIV protection was demonstrated in an experimentally challenged macaque model. The protection observed with the bNAb b12 was markedly reduced after the modification of the Fc domain, leading to impaired FcR binding (159). However, Fc-mediated effector functions might not be absolutely necessary to generate maximum protection, as was recently shown for the bNAb PGT121. In this study, a mutation impairing FcγR binding of PGT121 did not modify the protective effect of the bNAb (160).

## Antibody Capture of Infectious HIV Particles/Aggregation

The Fc domains of Abs can directly bind the virus, leading to the formation of virus/Ab aggregates. Ab inhibition by aggregation of a pathogen is a very basic inhibitory mechanism that results in a decrease in viral infectivity (161). The potential role of aggregation was reviewed recently (24, 146, 162).

Formation of the Ab/virus immune complex may hinder viral movement and impede viral replication by adhering the complex to mucus and restraining its transfer and transcytosis across mucosal epithelial cells (Figure 1). HIV aggregates will be trapped more efficiently than free virus particles (e.g., in the female reproductive tract, where there is abundant cervical mucus) (163). Moreover, immune aggregates may be retained efficiently in the mucus by binding of the Fc domain of IgG to mucins and specific binding in the vaginal tract to MUC16 (164). Analogous mechanisms may act on other mucosal surfaces, such as those in the gastrointestinal tract (165).

These findings suggest a direct inhibitory effect of HIV/Ab immune complex formation on HIV infectivity. Abs that retain HIV and hinder its diffusion through the epithelial barrier need to be better characterized to elucidate how they can be selectively induced at the mucosal site. We expect that these Abs recognize quaternary functional trimeric envelopes and non-functional Env spikes expressed on HIV particles. Therefore, the number of Abs able to form aggregates will be enlarged compared to that of NAbs, which will open up new opportunities to induce functional Abs with distinct epitope recognition by vaccination.

## Antibody-Mediated Complement Activation

The complement system is key to both innate and adaptive immunity, where it exerts multiple functions. Complement activation occurs through three distinct pathways (classical, alternative and lectin), which result in several types of antimicrobial activity, such as opsonization, inflammatory cell recruitment, cell lysis and virolysis [see review (24, 26, 166, 167)].

HIV has developed a sophisticated defense to protect itself by failing to bind complement proteins (168). Indeed, the gp120 Env does not bind complement (168). Moreover, during budding, HIV incorporates glycosyl phosphatidylinositol (GPI)-anchored CD55 and CD59 as well as transmembrane CD46, which are downregulatory molecules that inhibit complement-mediated damage to the virus (169). HIV also captures serum complement factor H, which plays a central role in protecting cells from complement by downregulating complement binding and in turn increases virulence (170–172). For these reasons, the use of primary isolates produced by primary cells is absolutely mandatory for *in vitro* studies of complement-mediated responses.

Moreover, bNAbs engineered to lack complement binding activity do not lose their protective effect, as shown in macaques challenged with SHIV (159), which is in contrast to those engineered to lack FcR. This finding suggests that complement may not be required for optimal *in vivo* Ab protection against infection. Nonetheless, in the RV144 vaccine trial that showed modest efficacy, complement activation induced by V1V2-specific Abs was stronger and more frequently detected compared to that in two related trials, VAX003 and VAX004, in which no significant protection was observed (173).

In contrast, complement and Ab may contribute to the enhancement of infection, as first described for Dengue virus (174). Binding of IgGs to FcRs induces the enhanced transcytosis of the virus at mucosal sites, although the exact mechanism is still unclear (Figure 1) (175). Moreover, whether this phenomenon has physiological relevance during HIV infection is still under debate (176, 177).

Collectively, complement components and their interactions with their cognate receptors are key to controlling adaptive immune responses, which provides insight into the use of complement components as novel drug targets. However, the relative contribution of complement to virolysis vs. viral enhancement in tissues and the periphery needs to be further investigated to understand its role in protection against HIV.

## Roles of Fc-Mediated Antibody Functions in Mucosal Tissues

Mucosal surfaces are the first entry site for HIV during transmission (Figure 1). Indeed, Langerhans cells (LCs), urethral macrophages and/or conventional DCs residing in mucosal tissues have been proposed to capture HIV (178–185) and further replicate or transfer the virus locally to potential HIV target cells, such as macrophages and CD4^+^ T cells (Figure 1). NK cells, macrophages and DCs, which are the effector cells involved in Fc-mediated Ab functions, are present in different mucosal tissues at different levels. Accordingly, mucosal effector cells may act through an Fc-mediated Ab response and be the first cells that modulate the early events of HIV transmission. The relative contribution of any of the aforementioned Fc-mediated Ab responses will depend on the frequency and distribution of the cells present within a given tissue and their FcR expression levels (3, 26, 182, 184, 186). Overall, FcR expression on cells and their affinities and binding profiles for Abs will directly impact Fc-mediated functions. Indeed, a comparison of penile, cervical and intestinal tissues showed that the expression profile of FcR on mucosal effector cells was reduced compared to that on blood cells, although the overall cell frequency was substantially different (187). Specifically, FcγRII^+^ DCs and macrophages were well-represented in all three tissues, whereas FcγRIII^+^ NK cells were rare only in the intestinal mucosa. We may imagine that Fc-mediated Ab function(s) may be less relevant in the blood circulation, where infected CD4^+^ T cells express very little FcγR on their surfaces (188).

Importantly, FcR expression varies according to the immune cell type and the localization and activation status. FcR-bearing cells, such as macrophages and DCs, predominate and tightly interact with tissues, which may facilitate their activity. Although resident NK cells express negligible levels of FcRs, mature circulating NK cells expressing high FcγRIII levels are rapidly recruited to the site of infection. Therefore, Fc-mediated inhibitory functions involving binding to FcγR on resident tissue cells may be of particular interest for the inhibition of mucosal transmission, although the exact mechanisms underlying FcR expression are not well-defined. Further studies on the potential role of Fc-bearing HIV targets and the involvement of Fc-mediated Ab inhibition at mucosal sites are needed to inform HIV vaccine strategies.

## Strategies to Induce Fc-Mediated Antibody Functions by Vaccination

Antibodies have now been proven to contribute to HIV protection and are therefore a central component of new vaccine strategies (189). As Fc-FcR interactions are able to generate powerful extraneutralizing Fc functions, these additional functions should be defined (159).

Numerous vaccine trials have already reported the induction of Fc-mediated functions. Vaccination with the gp120/CD4 mAb immune complex was found to be more efficient in inducing Fc-mediated adjuvant activity than that with gp120 alone. However, highly variable elements in the gp120 sequence limit the breadth of the responses to immune complex vaccines to a few HIV-1 isolates (190, 191). Moreover, vaccination with the SIVmac239-ΔNef virus induced an FcR-mediated inhibitory response that prevented founder virus entry and avoided local expansion (192). These results suggest protective Fc-mediated Ab function against transmitted/founder viruses at the mucosa surface. Another study demonstrated robust polyfunctional non-NAb responses, such as ADCC and ADCP, associated with protection against SIVmac251 challenge following vaccination with adenovirus serotype 26 (Ad26) vector priming/purified envelope (Env) glycoprotein boosting strategy in rhesus monkeys (151). In addition, a robust non-NAb response to the V1V2 region of the gp120 Env glycoprotein, which has been shown to exhibit Fc-mediated functions, was associated with a decreased risk of HIV acquisition in the RV144 clinical vaccine trial (193, 194). These different vaccine trials indicate that Abs displaying Fc-mediated functions participate in protection against HIV acquisition. This protection may be particularly efficient when several Fc-mediated activities are combined (158). Fc polyfunctionality increases over time following HIV infection and has recently been correlated with the further induction of neutralizing activity (195).

Therefore, we hypothesize that the induction of immune responses leading to Fc polyfunctionality may require the presentation of multiple antigens, similar to what induces bNAb production. Indeed, multiple antigens appear to be required for induction of bNAbs, as they appear following long periods of high viral replication in which there is continuous mutation of the viral Env protein. The general concept that the induction of an efficient humoral response, i.e., with bNAbs, and possibly Fc polyfunctionality will require high viral replication is sometimes misleading within the field. The correlation between an efficient Ab response and a high viral load provides a confusing dichotomist message regarding the potential role of Abs in protection against HIV acquisition. Accordingly, we may need a complex combination of immunogens to induce efficient Fc polyfunctional Abs as for induction of bNAbs. A more systematic analysis of Fc polyfunctional activity induced following vaccination is mandatory to understand the dynamics of the induction of functionally relevant Abs and to avoid switching to an immunodominant non-functional decoy Ab response.

To define the correlates between Ab profiles and protective functions, an integrative approach analyzing the “humoral Fc fingerprints” of vaccines was proposed. According to these principles, Chung et al. developed a unique approach called “Systems Serology” to retrospectively examine recent vaccine trials in humans to reveal features of immune complex composition underlying protective immunity to HIV (196). Using the “Systems Serology” approach, the protective humoral response signatures in vaccinated or naturally infected individuals in HIV can be defined. Moreover, systematic data production and application of machine learning approaches may identify distinct immunogenic regimens and Fc effector functions, allowing the selection of promising vaccine candidates (197).

These different studies provide insight into how to potentially induce Fc-mediated functions able to protect or control HIV infection via Fc-mediated antiviral activity (198, 199). New vaccine strategies aimed at directly inducing Fc-mediated activity should now be designed to improve the induction of potential functional activity in addition to highly desirable neutralizing activity.

## Summary and Conclusion

This review highlights the potential role of FcγR-mediated Ab immune functions besides neutralization in protection against HIV. Although the role of the Fc-mediated function of Abs lacking broadly neutralizing activity for HIV protection is still a matter of debate (160, 200), numerous independent studies now hint at their relevance for HIV inhibition. Above all, the specific Fc-mediated functions of non-NAbs are the only correlates of protection against infection observed in the RV144 vaccine trial conducted in Thailand (28, 29, 193, 201). However, no *in vivo* demonstration of the pertinence of non-NAbs for the prevention of HIV transmission is currently available. Dedicated experiments utilizing the NHP model may help dissect the role of Fc-mediated Ab responses and their relevance for the prevention of HIV infection. Nonetheless, to exert their function, these Abs must colocalize with the appropriate FcR-bearing cells at the site of infection. This scenario may occur during the early events of HIV mucosal transmission, when the virus crosses the epithelial barrier to infect the underlying target cells and further disseminate to other organs. Close contact between the virus, infected cells, Abs and effector cells in mucosal tissues may provide an ideal environment for Fc-mediated Ab functions to occur. We can also imagine that the prevalent and relevant Fc-mediated Ab function(s) may differ according to the infection route and mucosal type involved (i.e., genital or intestinal mucosa). An important hurdle for HIV vaccine designers will be to determine how to induce high concentrations of Abs with Fc-mediated functions directly within the mucosal sites of exposure, which is a challenge that may equal or be greater than that of inducing bNAbs (202).

## Author Contributions

BS wrote the first draft of the manuscript. BS, SD, RC, GS, and CM revised the manuscript. All authors listed have made a substantial, direct and intellectual contribution to the work, read, and approved the final manuscript.

### Conflict of Interest

The authors declare that the research was conducted in the absence of any commercial or financial relationships that could be construed as a potential conflict of interest.
